# Prevalence and correlates of vascular plaques and high intima thickness in a group of patients with high cardiovascular risk in Cameroon

**DOI:** 10.11604/pamj.2022.41.80.31944

**Published:** 2022-01-28

**Authors:** Liliane Mfeukeu-Kuate, Emerentia Eho Walinjom, Maggy Mbede, Joshua Njimbuc Walinjom, Ba Hamadou, Ahmadou Musa Jingi, Mazou Temgoua, Jan René Nkeck, Jean Jacques Noubiap, Samuel Kingue

**Affiliations:** 1 Faculty of Medicine and Biomedical Sciences, University of Yaoundé I, Yaoundé, Cameroon,; 2 Medical and Cardiology Unit, Yaoundé Central Hospital, Yaoundé, Cameroon,; 3 Faculty of Health Sciences, The University of Bamenda, Bamenda, Cameroon,; 4 Centre for Heart Rhythm Disorders, The University of Adelaide, Adelaide, Australia,; 5 Medical and Cardiology Unit, Yaoundé General Hospital, Yaoundé, Cameroon

**Keywords:** Carotid artery, femoral artery, intima-media thickness, plaques, cardiovascular risk factors, sub-Saharan Africa

## Abstract

**Introduction:**

carotid and femoral intima-media thickness (IMT) and atherosclerotic plaques are considered as markers of generalized atherosclerosis and as independent predictors of cardiovascular events and mortality. This study aimed to determine the prevalence and correlates between carotid and femoral intima-media thickness and plaques in patients with major cardiovascular risk factors (CVRFs).

**Methods:**

we carried out a cross-sectional study at the Yaoundé Central Hospital between December 2017 and May 2018. B-mode ultrasound was used to assess for the presence of plaques and also measure the IMT at the carotid and femoral arteries in patients with CVRFs. Logistic regression analysis was performed to examine the association between ultrasound findings (presence of plaques or IMT > 0.9mm) and cardiovascular risk factors. A p-value <0.05 was considered significant.

**Results:**

amongst the 71 patients, 43.7% were male and 56.3% were female. The mean age was 61.6 ± 8.4 years and ranged from 40 to 75 years. Thirty-nine (54.9%) participants had carotid atherosclerotic plaques and 33 (46.5%) participants had femoral artery plaques. The plaque burden was higher in the carotid arteries. Plaques at one or more artery sites were seen in 67.6% of participants. An IMT ≥ 0.9 mm was seen in only 1.4 to 2.8% of participants. In the multivariable analysis using binary logistic regression, age > 50 years (males) or 60 years (females) (aOR: 11.3 [95% CI: 2.2 - 56.8], p=0.002) and presence of dyslipidemia (aOR: 3.6 [95% CI: 1.2 - 11], p=0.043) were associated with carotid artery plaques, while presence of dyslipidemia (aOR: 4.8 [95% CI1.8 - 13.3], p=0.004) and high cardiovascular risk profile (10-year risk> 20%) (aOR: 4.2 [95% CI: 1.2 - 13.2], p=0.0495) were associated with femoral artery plaques.

**Conclusion:**

plaques were more frequent than an IMT > 0.9 mm, with a higher plaque burden in the carotid arteries. Plaques were associated with advanced age, dyslipidemia, and a high cardiovascular risk profile.

## Introduction

Cardiovascular disease (CVD) is a major cause of disability and premature death worldwide. The most common underlying cause is atherosclerosis, which develops over many years and is usually advanced by the time symptoms occur, generally in middle age [1]. An estimated 17.5 million people died from CVD in 2015, representing 31% of all deaths globally. This statistic is expected to grow to more than 23.6 million by 2030. Over three-quarters of CVD deaths take place in low and middle-income countries [2]. To prevent death and morbidity from CVD, there is great interest in the early identification of asymptomatic patients at high risk who will benefit from more intensive lifestyle and therapeutic intervention to prevent disability and death, improve quality of life and reduce the global healthcare cost.

Accurate and cost-effective identification of subjects at risk remains a challenge [3]. Ultrasound screening for pre-symptomatic peripheral atherosclerosis (ATS) is increasingly recognized as a useful method for enhancing the detection of high-risk subjects beyond cardiovascular risk factors (CVRF) [4]. B-mode ultrasound is a non-invasive method of examining the walls of peripheral arteries and provides measures of intima-media thickness (IMT) and the presence of plaques [5]. Carotid IMT and femoral IMT can be considered both as surrogate markers of generalized atherosclerosis and as independent predictors of cardiovascular events and mortality. The presence of plaques has also been associated with incident stroke or myocardial infarction and cardiovascular mortality [4,6]. Studies have reported some inconsistent associations between a range of cardiovascular risk factors (smoking, blood pressure, elevated blood cholesterol), IMT, and clinical disease. These studies have also highlighted the importance of the presence and severity of arterial wall plaque as determinants of adverse cardiovascular even events [7-11]. Since thicker IMT bifurcation and bulb, origin values tend to occur in people who also have plaques, presence or absence of plaque, and not IMT may be the more relevant indicator of early atherosclerosis [5].

Few data exist on subclinical markers of CVD in Cameroon because most patients are often diagnosed at the advanced stages of vascular disease and most of them may not have symptoms at its onset or might have atypical symptoms. Little is known about the associations between increased IMT or plaques and cardiovascular risk factors in our milieu. Hence, we sought to determine the association between carotid and femoral intima-media thickness, plaques, and cardiovascular risk factors at the Yaounde Central Hospital.

## Methods

### Study design and setting

This was a hospital-based cross-sectional study conducted from December 2017 to May 2018 at the Cardiology Unit of the Yaoundé Central Hospital (YCH). This hospital serves as one of the teaching hospitals of the University of Yaoundé 1. It has a catchment population of over 2 million inhabitants. Yaoundé is the political capital of Cameroon, the Central African sub-region.

### Study participants

We included consenting adults of both sexes aged ≥40 years with at least two major cardiovascular disease risk factors. We excluded patients with significant lower limb edema and those with incomplete data.

### Procedure and measurements

Eligible participants were seen twice. In phase 1, they underwent a focused interview, physical examination, assessment of ankle-brachial index (ABI), and blood drawn for lipid profile assay. Vascular ultrasound was carried on in phase 2. Socio-demographic data collected were; age, sex, and profession. We collected information on the history of hypertension, diabetes, chronic kidney disease, tobacco use (quantified as packet-years for smokers), family history of stroke, and coronary heart disease in first-degree relatives. A sedentary lifestyle was assessed as physical activity less than 3 days of 30 minutes equivalence of brisk walking daily. Intermittent claudication was assessed using the Edinburgh claudication questionnaire. Eligible participants were then examined. Blood pressure was measured on the right arm with a validated electronic device (OMRON®) using a standard procedure (5 minutes of rest in the sitting position). The average of the two measurements was considered. The weight (kg) was measured with an electronic scale balance and height (m) with a stadiometer. These values were used to calculate the body mass index (BMI) using Quetelet´s formula. The waist circumference (cm) was measured using a flexible tape to assess for central obesity. Participants then underwent a detailed physical examination to look for vascular murmurs and signs of peripheral artery disease. Venous blood was then drawn after 9 hours of fasting to assess the lipid profile using a standard biochemical procedure.

### Ankle-brachial index (ABI) measurement

ABI was measured using a continuous-wave Doppler machine (Edam Sonotrax vascular Doppler) with a high-frequency probe (10MHz). The blood-pressure cuff was placed on the patient´s right or left arm. The brachial pulse was palpated. The gel was applied at the site where the pulse was felt, and a Doppler signal was obtained by placing the probe at a 60-degree angle toward the patient´s head. The cuff was then inflated rapidly to 20 mm Hg above the point of cessation of brachial-artery flow, then the blood-pressure cuff was slowly deflated to note the systolic value. The gel was wiped from the patient´s skin and the procedure was repeated on the other arm. After measuring the systolic blood pressure in the arms, the cuff was placed just above the ankle on the right or left leg. The Doppler probe was placed on the palpable dorsalis pedis pulse or on the site that produces the best arterial Doppler signal from the dorsalis pedis artery. Once again, the blood-pressure cuff was inflated to 20mm Hg above the level at which flow ceases, then the cuff was deflated slowly and the systolic pressure was noted (the pressure at which you first hear the flow from the dorsalis pedis artery). The procedure was repeated for the posterior tibial artery. Then repeated for the contralateral leg to obtain the systolic pressure from both the dorsalis pedis and posterior tibial arteries. An ankle-brachial index was obtained by dividing the average of the systolic pressures measured from the dorsalis pedis artery and the posterior tibial artery on each limb by the systolic blood pressure in each arm. The lower of the two values of the ankle-brachial index (ABI) was considered.

### Ultrasound measurements

High-resolution B-mode and color Doppler mode ultrasound were performed with a portable system (Sonoscape S50) connected to an 8-10 MHz linear array transducer. All measurements were performed by the same experienced sonographer. The right and left carotid and femoral arteries were examined in all participants. Carotid arteries were examined from the supraclavicular fossa to the sub-mandibular angle, including the common carotid artery (CCA), the bulb, and the origin of the internal and external carotid arteries. The common femoral arteries (CFA) were scanned from the level of the inguinal ligament to the end of the adductor canal.

### Intima media thickness measurement

High-resolution B-mode ultrasound and colored Doppler mode were used to measure the IMT on both the far-wall and near-wall of the common carotid and common femoral arteries. The IMT was measured manually. Intima-media thickness, deïned as the distance from the leading edge of the lumen-intima interface to the leading edge of the media-adventitia interface, was measured on both CCAs and CFAs on a distance of 1cm before the flow divider. Optimal longitudinal frames of these segments were frozen in late diastole before analysis. IMT was measured from the far wall and the near wall of both the CCAs and CFAs. The measurements obtained from the far wall were used to calculate the mean IMT. Several measurements of IMT were taken manually on a 10mm segment and the mean IMT was calculated. Finally, the mean values on each side were averaged to obtain a single mean variable at the carotid and femoral levels. IMT was measured only for those who did not have plaques in any of the artery sites (CCAs and CFAs).

### Plaque measurement

Plaques are focal structures encroaching into the arterial lumen of at least 0.5 mm or 50% of the surrounding IMT value or demonstrate a thickness > 1.5 mm as measured from the intima-lumen interface to the media-adventitia interface. Plaques were identified on both the near and the far walls in all the above described arterial segments by a thorough transversal and longitudinal scanning. After detection, plaques were scanned in the best longitudinal view perpendicularly crossing the most prominent part of the lesions and scans frozen in late diastole. Plaque thickness was considered as the distance between the plaque-lumen interface and the plaque-adventitia interface of the thickest plaque visualized on each site.

### Cardiovascular risk assessment with the WHO/ISH cardiovascular risk prediction chart for AFR D

At the end of the second phase, we estimated the patient´s level of cardiovascular risk with the WHO/ISH risk prediction charts for the AFR D sub-region. These subjects were classified into three levels of cardiovascular risk; Low risk (risk < 10%), intermediate-risk (risk from 10% to 20%), and high risk (risk > 20%).

### Definitions

Intima-media thickness (IMT) ≥ 0.9mm was considered abnormal [12]. Atherosclerotic plaque is focal structures encroaching into the arterial lumen of at least 0.5 mm or 50% of the surrounding IMT value or demonstrates a thickness >1.5 mm as measured from the intima-lumen interface to the media-adventitia interface [13]. Lower extremity peripheral artery disease (PAD) was defined as an ABI <0.90. Stroke was defined as documented cerebrovascular disease confirmed by a CT scan. Smoking was considered as someone who was currently smoking or who had stopped for less than one year [14]. Physically active individuals were defined as those who do 150 minutes of moderate-intensity aerobic physical activity throughout the week or do at least 75 minutes of vigorous-intensity aerobic physical activity throughout the week or an equivalent combination of moderate- and vigorous-intensity activity [15]. Hypertension was considered present if any of the following conditions were met: patient diagnosed in a health facility and/or reported use of a medication for hypertension and/or systolic blood pressure ≥ 140 mmHg, diastolic blood pressure ≥ 90 mmHg [16]. Diabetes was considered present if any of the following conditions were met: patient diagnosed in a health facility and/or reported use of a medication for diabetes mellitus or fasting blood sugar ≥ 1.26 g/L [17]. The body mass index (BMI) was calculated as the ratio of the weight (kg) to the square of height (m). According to the WHO criteria, the individuals were considered: underweight if BMI < 18 kg/m^2^, normal if BMI ≥ 18 and < 25 kg/m^2^, overweight if BMI ≥ 25 and < 30 kg/m^2^; obese if BMI ≥ 30 kg/m^2^ [18]. Abdominal obesity was defined according to the National Cholesterol Education Program (NCEP) by a waist circumference greater than 102 cm in men and 88 cm in women [19]. Lipid disorders were defined as follows: High TC > 200mg/dL, Low HDL-C < 40mg/dL, High LDL-C > 130 mg/dL, High TG > 150 mg/dL. Dyslipidemia was considered present if any one of the above lipid parameters were abnormal [19].

### Sample size

We used Cochran´s formula to estimate the minimum sample size for this study. With an expected prevalence of PAD of 10%, for an 80% power to detect significant associations or differences, and a 5% error margin, the minimum sample size for this study was 62 participants.

### Data analysis

The data were analyzed with Epi-Info version 7. We have presented discrete variables as frequencies and percentages according to sex. We have presented continuous variables as means (standard deviation). Univariable and multivariable binary logistic regression analysis was performed to examine the association between ultrasound findings (presence of plaques or IMT ≥ 0.9mm) and cardiovascular risk factors (predictor variables). Variables with a p-value < 0.2 in univariable analysis were considered in the multivariable analysis, adjusting for age and gender. A p-value < 0.05 was considered significant for the observed associations.

### Ethical considerations

Ethical clearance was obtained from the Ethical Committee of the Faculty of Medicine and Biomedical Sciences, University of Yaounde I, and administrative authorization from the director of the Yaounde Central Hospital. All participants provided signed informed consent.

## Results

### General characteristics

A total of 110 participants were approached and 39 were excluded for incomplete data. Of the 71 participants included in the study, 31 (43.7%: [95% CI: 31.9 - 56]) were male, and 40 (56.3%: [95% CI: 44.1 - 68.1]) were female. The mean age was 61.6 ± 8.4 years and ranged from 40 to 75 years.

### Cardiovascular risk profile

The clinical and biochemical characteristics of the study population are shown in Table 1. The most frequent vascular risk factors (positive medical history) were hypertension (80.3%), advanced age (71.8%), dyslipidemia (50.3%), and diabetes (47.9%). Tobacco use, alcohol consumption, and global obesity were significantly higher in males. Central obesity was significantly higher in females. Males also had significantly high rates of dyslipidemia and high cardiovascular risk. A high cardiovascular risk profile was seen in 15 (21.1%, [95% CI: 12.3 - 32.4]) participants. Peripheral artery disease (ABI < 0.9) was seen in 5 (7%: [95% CI: 2.3 - 15.7]) patients. Intermittent claudication was seen in 6 (8.5%, [95% CI: 3.2 - 17.5]), and this was predictive of PAD (OR: 35.5, [95% CI: 3.7 - 265.4], p<0.001).

**Table 1:** clinical and biochemical characteristics of the study population

Variable	Overall, n (71)	Male, n (31)	Female,n (40)	p-value
Age (years), mean (SD)	61.6 (8.4)	62.2 (8)	61.2 (8.8)	0.616
Male sex, n (%)	-	43.7	56.3	-
Medical history				
Hypertension, n (%)	57 (80.3)	25 (80.7)	32 (80)	0.942
Stroke, n (%)	15 (21.1)	9 (29.3)	6 (15)	0.147
Diabetes, n (%)	34 (47.9)	14 (45.2)	20 (50)	0.690
Tobacco use, n (%)	10 (14.1)	10 (32.3)	0 (0)	<0.001
Alcohol use, n (%)	28 (39.4)	17 (54.8)	11 (27.5)	0.020
Intermittent claudication, n (%)	6 (8.5)	2 (6.5)	4 (10)	0.602
**Physical findings**				
BMI, mean (SD)	29.1 (5.9)	30.6 (7.1)	27.3 (3.1)	0.077
**BMI class**				
Normal, n (%)	16 (22.5)	6 (19.4)	10 (25)	0.578
Overweight, n (%)	24 (33.8)	5 (16.1)	19 (47.5)	0.006
Obese, n (%)	31 (43.7)	20 (64.5)	11 (27.5)	0.002
Waist circumference (cm), mean (SD)	93.3 (14.4)	94.5 (15.8)	93.5 (12.5)	0.706
Waist circumference > 80 or 94 cm, n (%)	46 (64.8)	15 (48.4)	31 (77.5)	0.012
Systolic BP (mmHg), mean (SD)	139.4 (18.9)	140.5 (19.1)	138.6 (19)	0.939
Systolic BP ≥ 140 mmHg, n (%)	35 (49.3)	13 (41.9)	22 (55)	0.277
Diastolic BP (mmHg), mean (SD)	82.8 (11.2)	84.1 (11.8)	81.8 (10.8)	0.667
Diastolic BP ≥ 90 mmHg, n (%)	20 (28.2)	8 (25.8)	12 (30)	0.698
Pulse pressure (mmHg), mean (SD)	56.6 (15.8)	56.3 (15.2)	56.8 (16.4)	0.926
Pulse pressure ≥ 65 mmHg, n (%)	17 (23.9)	7 (22.6)	10 (25)	0.816
Ankle Brachial Index, mean (SD)	1.02 (0.1)	1.01 (0.1)	1.03 (0.1)	0.165
Ankle Brachial Index < 0.9, n (%)	5 (7.04)	3 (9.7)	2 (5)	0.446
**Biochemical data**				
Total Cholesterol (mg/dL), mean (SD)	163.4 (38.9)	164 (38.3)	163 (39.7)	0.655
Total Cholesterol > 200 mg/dL, n (%)	9 (12.7)	5 (16.1)	4 (10)	0.447
HDLc (mg/dL), mean (SD)	45.6 (14.6)	41.1 (14.5)	49 (13.8)	0.016
HDLc < 40 or 50 mg/dL, n (%)	33 (46.5)	14 (45.2)	19 (47.5)	0.848
LDLc cholesterol (mg/dL), mean (SD)	97.5 (30.2)	107.9 (28.7)	89.5 (29.1)	0.002
LDL cholesterol > 100 mg/dL, n (%)	26 (36.6)	17 (54.8)	9 (22.5)	0.005
TC/HDLc ratio, mean (SD)	4.1 (2.2)	4.6 (2.2)	3.7 (2.1)	0.041
TC/HDLc ratio > 5, n (%)	15 (21.1)	10 (32.3)	5 (12.5)	0.044
Dyslipidemia, n (%)	33 (46.5)	18 (58.1)	15 (37.5)	0.087
**Cardiovascular Risk profile**				
Low risk, n (%)	26 (36.6)	10 (32.3)	16 (40)	0.507
Intermediate risk, n (%)	30 (42.3)	10 (32.3)	20 (50)	0.403
High risk, n (%)	15 (21.1)	11 (35.5)	4 (10)	0.001

ABI: ankle-brachial index; aOR: adjusted odds ratio; BMI: body mass index; BP: blood pressure; HDLc: high-density lipoprotein cholesterol; LDLc: low-density lipoprotein cholesterol; SD: standard deviation; TC: total cholesterol

### Prevalence of vascular plaques and high intima thickness

The echocardiographic data are shown in Table 2. Vascular plaques were the most frequent echographic findings in both carotid (54.9%, [95% CI: 42.7 - 66.8]) and femoral arteries (46.5%: [95% CI: 34.6 - 58.7]). An IMT > 0.9 mm was less frequent (1.4 to 2.8%) in both major arteries. The plaque burden (number and size) was higher in the carotid than in the femoral vessels. Plaques at one or more artery sites were seen in 48 (67.6%) participants. Plaque at one site was predictive of the other site (OR: 4.1, [95% CI: 1.5 - 11.2], p=0.005).

**Table 2:** carotid and femoral echographic features

Variable	Overall, n (71)	Male, n (31)	Female, n (40)	P value
**Carotid Artery**				
Common carotid IMT (mm), mean (SD)	0.56 (0.2)	0.58 (0.2)	0.55 (0.2)	0.586
Common carotid IMT > 0.9 mm, n (%)	1 (1.4)	1 (3.2)	0 (0)	0.258
Carotid plaques (yes), n (%)	39 (54.9)	20 (64.5)	19 (47.5)	0.156
Number of carotid plaques, mean (SD)	0.94 (1.1)	1.0 (1.2)	0.9 (1.1)	0.892
Carotid plaque thickness (mm), mean (SD)	0.88 (0.9)	0.79 (0.85)	0.94 (0.98)	0.567
**Femoral Artery**				
Femoral artery IMT (mm), mean (SD)	0.45 (0.2)	0.45 (0.2)	0.44 (0.2)	0.444
Femoral artery IMT > 0.9 mm, n (%)	2 (2.8)	2 (5)	0 (0)	0.156
Femoral artery plaques (yes), n (%)	33 (46.5)	17 (54.8)	16 (40)	0.218
Number of femoral artery plaques, mean (SD)	0.7 (0.8)	0.77 (0.85)	0.65 (0.83)	0.502
Femoral artery plaque thickness (mm), mean(SD)	0.76 (0.95)	0.87 (1.03)	0.67 (0.88)	0.338

IMT: intima-media thickness; SD: standard deviation

### Correlates of vascular plaques and high intima thickness

The factors associated with the occurrence of plaques are shown in Table 3. In univariable analysis, advanced age (> 50 years in males and > 60 years in females), presence of dyslipidemia, and high cardiovascular risk were associated with the presence of carotid artery plaques. ABI, dyslipidemia and high cardiovascular risk were associated with femoral artery plaques. Patients with abdominal obesity appeared less likely to have carotid artery plaques. In the multivariable analysis using binary logistic regression, age > 50 years (males)/60 years (females) (aOR: 11.3, p=0.002) and presence of dyslipidemia (aOR: 3.6, p=0.043) were associated with carotid artery plaques, while dyslipidemia (aOR: 4.8, p=0.004) and a high cardiovascular risk profile (aOR: 4.2, p=0.0495) were associated with femoral artery plaques (Table 4).

**Table 3:** factors associated with the occurrence of carotid and femoral plaques from univariable logistic regression analysis

Variable	N (%)	Carotid plaques		N (%)	Femoral plaques	
		OR (95% CI)	p-value		OR (95% CI)	p-value
**Male gender**						
Yes	20 (64.5)	2.0 (0.8-5.3)	0.153	17 (54.8)	1.8 (0.7-4.7)	0.214
No	19 (47.5)	1		16 (40)	1	
**Age > 50 years♂ or 60 years♀**						
Yes	37 (66.1)	12.7 (2.6-62)	0.0003	27 (48.2)	1.4 (0.4-4.5)	0.571
No	2 (13.3)	1		6 (40)	1	
**Hypertension**						
Yes	32 (56.1)	1.3 (0.4-4.1)	0.679	29 (50.9)	2.6 (0.7-9.2)	0.151
No	7 (50)	1		4 (28.6)	1	
**Stroke**						
Yes	10 (66.7)	1.9 (0.6-6.2)	0.304	7 (46.7)	1.01 (0.3-3.2)	0.987
No	29 (51.8)	1		26 (46.4)	1	
**Diabetes**						
Yes	16 (47.1)	0.5 (0.2-1.4)	0.201	14 (41.2)	0.7 (0.3-1.7)	0.391
No	23 (62.2)	1		19 (51.4)	1	
**Tobacco**						
Yes	6 (60)	1.3 (0.3-5)	1	6 (60)	1.9 (0.5-7.4)	0.497
No	33 (54.1)	1		27 (44.3)	1	
**Intermittent claudication**						
Yes	3 (50)	0.8 (0.2-4.3)	1	4 (66.7)	2.5 (0.4-14.5)	0.407
No	36 (55.4)	1		29 (44.6)	1	
Obesity						
Yes	11 (45.8)	0.6 (0.2-1.6)	0.271	9 (37.5)	0.6 (0.2-1.6)	0.278
No	28 (55.6)	1		24 (51.1)	1	
**Waist Circumference > 80 or 94 cm**						
Yes	21 (45.7)	0.3 (0.1-0.9)	0.033	20 (43.5)	0.7 (0.3-1.9)	0.492
No	18 (72)	1		13 (52)	1	
**Systolic BP ≥ 140mmHg**						
Yes	20 (57.1)	1.2 (0.5-3)	0.712	17 (48.6)	1.2 (0.5-3)	0.727
No	19 (52.8)	1		16 (44.4)	1	
**Diastolic BP ≥ 90 mmHg**						
Yes	13 (65)	1.8 (0.6-5.2)	0.286	12 (60)	2.1 (0.8-6.2)	0.153
No	26 (51)	1		21 (41.2)	1	
**Pulse pressure ≥ 65mmHg**						
Yes	10 (58.2)	1.2 (0.4-3.7)	0.711	7 (41.2)	0.8 (0.3-2.3)	0.615
No	29 (53.7)	1		26 (48.2)	1	
**ABI < 0.9 mm**						
Yes	4 (80)	3.5 (0.4-33.4)	0.369	5 (100)	NA	0.012
No	35 (53)	1		28 (42.4)	1	
**Total cholesterol > 2g/L**						
Yes	6 (66.7)	1.8 (0.4-7.7)	0.499	6 (66.7)	2.6 (0.6-11.3)	0.287
No	33 (53.2)	1		27 (43.6)	1	
**HDLc < 0.4 or 0.5 g/L**						
Yes	20 (60.6)	1.5 (0.6-4)	0.370	18 (54.6)	1.8 (0.7-4.7)	0.204
No	19 (50)	1		15 539.5)	1	
**LDLc > 1g/L**						
Yes	18 (69.2)	2.6 (0.9-7.1)	0.066	16 (61.5)	2.6 (1-7.1)	0.053
No	21 (46.7	1		17 (37.8)	1	
**Dyslipidemia**						
Yes	23 (69.7)	3.2 (1.2-8.5)	0.020	22 (66.7)	4.9 (1.8-13.5)	0.002
No	16 (42.1)	1		11 (28.95)	1	
**TC/HDLc ratio > 5**						
Yes	9 (60)	1.3 (0.4-4.1)	0.657	10 (66.7)	2.9 (0.9-9.5)	0.078
No	30 (53.6)	1		23 (41.1)	1	
**High Cardiovascular risk**						
Yes	12 (80)	4.3 (1.1-16.9)	0.040	11 (73.3)	4.3 (1.2-15)	0.023
No	27 (48.2)	1		22 (39.3)	1	

ABI: ankle-brachial index; OR: odds ratio; BP: blood pressure; HDLc: high-density lipoprotein cholesterol; LDLc: low-density lipoprotein cholesterol; TC: total cholesterol

**Table 4:** determinants of the occurrence of carotid and femoral plaques from multivariable logistic regression analysis

Variable	Carotid plaques		Femoral plaques	
	aOR (95% CI)	p-value	aOR (95% CI)	p-value
Male gender	1.4 (0.5-4)	0.748	1.8 (0.7-4.7)	0.378
Age > 50 years ♂ or 60 years ♀	11.3 (2.2-56.8)	0.002	1.2 (0.4-3.9)	0.973
Hypertension	0.6 (0.2-2.4)	0.697	2.5 (0.7-8.9)	0.282
Waist Circumference >80cm♀ or94 cm♂	0.4 (0.1-1.5)	0.311	0.9 (0.3-2.8)	0.985
Diastolic BP ≥ 90 mmHg	3.4 (0.8-13.7)	0.146	2.3 (0.8-6.7)	0.215
ABI < 0.9 mm	2.1 (0.2-21.4)	0.888	NA	
Total cholesterol > 2g/L	1.3 (0.3-5.5)	0.950	2.5 (0.6-10.8)	0.378
HDLc < 0.4 or 0.5 g/L	2.0 (0.6-7)	0.314	1.9 (0.7-4.9)	0.288
LDLc > 1g/L	2.6 (0.8-8)	0.188	2.6 (0.95-6.9)	0.106
Dyslipidemia	3.6 (1.2-11)	0.043	4.8 (1.8-13.3)	0.004
TC/HDLc ratio > 5	1.1 (0.3-3.5)	0.752	2.7 (0.8-9)	0.159
High Cardiovascular risk	3.5 (0.8-16.2)	0.182	4.2 (1.2-13.2)	0.049

ABI: ankle-brachial index; aOR: adjusted odds ratio; BP: blood pressure; HDLc: high-density lipoprotein cholesterol; LDLc: low-density lipoprotein cholesterol; TC: total cholesterol

## Discussion

We carried out this cross-sectional study to assess the occurrence and determinants of vascular plaques and an IMT > 0.9 mm in a group of patients with at least two vascular risk factors in a sub-Saharan Africa setting. Vascular plaques were the most frequent with a higher burden (number and size) in the carotid arteries. These were associated with advanced age, dyslipidemia, and a high vascular risk profile. An IMT > 0.9 mm was less frequent in both vascular sites.

To prevent death and morbidity from cardiovascular disease, there is great interest in the early identification of asymptomatic patients at high risk who could benefit from more intensive lifestyle and therapeutic interventions. However, the accurate and cost-effective identification of patients at risk is still a challenge [3]. Ultrasound screening for pre-symptomatic peripheral atherosclerosis (ATS) is increasingly recognized as a useful tool for enhancing the detection of high-risk patients beyond cardiovascular risk factors (CVRF) [4]. However, this technology is not widely and readily available in our setting. Several studies have shown that the older population has increased IMT and the presence of plaques [20]. Hypertension, age, and dyslipidemia were the most prevalent cardiovascular risk factors in this group of patients. This is similar to that reported by Menanga *et al*. [21]. Plaques were most common at the bulb of the carotid artery. Our results are similar to those reported by Tasneem *et al*. [22]. Atherosclerotic plaques generally develop at branch Ostia and bifurcation of the common carotid artery into the external and internal carotid artery. The ostium of the internal carotid artery is mostly affected, involving the posterior wall of the carotid sinus. It also extends into the distal common carotid artery. Besides the general risk factor for atherosclerosis, fluid dynamics and vessel geometry also play a role in the development of atherosclerosis. Hemodynamic forces at the carotid bifurcation have a role in the localization of intimal thickening. In both in vivo and in vitro studies, it has been reported that disturbed flow and low-shear conditions produce endothelial dysfunction [23]. Endothelial cell dysfunction induces the accumulation of inflammatory cells, migration and proliferation of smooth muscle cells, and release of various cytokines and chemokines, which cause carotid plaques and stenosis [24]. In this study, 7% of participants had PAD. This was only predictive of femoral plaques and not carotid plaques. These findings suggest that ABI might not be not an accurate screening tool for diagnosing early atherosclerosis. Therefore, vascular ultrasound is superior in detecting early atherosclerosis. Our findings are similar to that reported by Flanigan *et al*. who found that superficial femoral artery duplex ultrasound is an accurate screening tool and can be utilized in screening protocols in place of the time-honored ABI [25].

We found that dyslipidemia and advanced age were the most frequent cardiovascular risk factors in those with plaques both at the carotid and femoral artery levels. The association between conventional risk factors and IMT has been established in previous studies. Hypertension, diabetes mellitus, smoking, higher levels of systolic blood pressure (SBP), and dyslipidemia are associated with increased IMT and atherosclerotic plaques [4,26]. Arterial remodeling processes in hypertension and carotid atherosclerosis involve pathological changes, including the inflammatory response and damage caused to endothelial cells in the arterial vascular wall. Hypertension and atherosclerosis have a mutually reinforcing relationship. The prominent pathological changes in hypertension are vascular remodeling and hypertrophy. In the gradual development of hypertension, atherosclerosis is an important cause of vascular remodeling in small blood vessels, which further damages the target organs. Meanwhile, the increase in fluid shear stress also accelerates atherosclerosis in large vessels. The consequence of hypertension is systemic arterial atherosclerosis. Elevated blood pressure is one factor that promotes atherosclerosis [27]. We found that IMT, plaques, and the number of patients with stroke showed a statistically significant increase across the groups as we move from low risk to high risk at the carotid arteries. This indicates a higher frequency of atherosclerotic lesions in patients at high risk as compared with those at lower cardiovascular risk. There was a significant correlation between age and mean intima thickness. Yerly *et al*. reported similar findings [4]. Aging is associated with geometrical changes in carotid arteries (elastic arteries) independent of risk factors like blood pressure. Atherosclerosis increases with age and could lead to increase IMT. Finally, a greater proportion of subjects diagnosed with stroke or PAD were in the high-risk group and they had abnormal ultrasound findings (IMT and Plaques).

This study has some limitations. It is a hospital-based study in patients with cardiovascular risk factors. Our findings do not reflect on the burden of disease in the community. Diabetes was ascertained only from the history. Sub-clinical cases of diabetes must have been missed which could change the risk profile of the participants. This study was limited to the traditional risk factors. Novel risk factors such as ultrasensitive C-reactive protein (CRP), homocysteine were not studied. Even though the minimal sample size was attained, our study appeared underpowered to capture significant associations of vascular risk factors and the presence of vascular plaques and a high IMT. The very small number of participants with high IMT did not permit us to carry further statistical analysis to look for the associated factors. Despite these limitations, this is the first study in our setting to assess the occurrence and determinants of vascular plaques and IMT > 0.9 mm.

## Conclusion

In this group of patients with at least two vascular risk factors in a sub-Saharan Africa setting, atherosclerotic vascular plaques were more frequent than an IMT > 0.9 mm, with a higher plaque burden in the carotid than femoral arteries suggesting a higher propensity to stroke than PAD. Plaques were associated with advanced age, dyslipidemia, and a high global cardiovascular risk profile. More studies are needed with higher sample size and including other novel vascular risk factors.

### What is known about this topic

- *Atherosclerosis is the most common underlying cause of Cardiovascular Disease, which develops over many years and is usually advanced by the time symptoms occur, generally in middle age;*
- *Little is known on the burden and associations between increased intima-media thickness or plaques and cardiovascular risk factors in sub-Saharan Africa*.

### What this study adds

- *In this group of patients with at least two vascular risk factors in a sub-Saharan Africa setting, atherosclerotic vascular plaques were more frequent than an Intima-Media-Thickness > 0.9 mm;*
- *The plaque burden was higher in the carotid than femoral arteries suggesting a higher propensity to stroke than peripheral artery disease*.
